# The Episode of Genetic Drift Defining the Migration of Humans out of Africa Is Derived from a Large East African Population Size

**DOI:** 10.1371/journal.pone.0097674

**Published:** 2014-05-20

**Authors:** Nuha Elhassan, Eyoab Iyasu Gebremeskel, Mohamed Ali Elnour, Dan Isabirye, John Okello, Ayman Hussien, Dominic Kwiatksowski, Jibril Hirbo, Sara Tishkoff, Muntaser E. Ibrahim

**Affiliations:** 1 Department of Molecular Biology, Institute of Endemic Diseases, University of Khartoum, Khartoum, Sudan; 2 Department of Biology, Eritrea Institute of Technology, Mai-Nefhi, Eritrea; 3 Department of Biochemistry, Makerere University, Kampala, Uganda; 4 Welcome Trust Centre for Human Genetics, University of Oxford, Oxford, United Kingdom; 5 Department of Genetics and Biology, School of Medicine and School of Arts and Sciences, University of Pennsylvania, Philadelphia, Pennsylvania, United States of America; Ben-Gurion University of the Negev, Israel

## Abstract

Human genetic variation particularly in Africa is still poorly understood. This is despite a consensus on the large African effective population size compared to populations from other continents. Based on sequencing of the mitochondrial Cytochrome C Oxidase subunit II (MT-CO2), and genome wide microsatellite data we observe evidence suggesting the effective size (*Ne*) of humans to be larger than the current estimates, with a foci of increased genetic diversity in east Africa, and a population size of east Africans being at least 2-6 fold larger than other populations. Both phylogenetic and network analysis indicate that east Africans possess more ancestral lineages in comparison to various continental populations placing them at the root of the human evolutionary tree. Our results also affirm east Africa as the likely spot from which migration towards Asia has taken place. The study reflects the spectacular level of sequence variation within east Africans in comparison to the global sample, and appeals for further studies that may contribute towards filling the existing gaps in the database. The implication of these data to current genomic research, as well as the need to carry out defined studies of human genetic variation that includes more African populations; particularly east Africans is paramount.

## Introduction

The fact that Africa is the cradle of modern humans and the scene of its major biological and demographic events is not of much dispute. This is manifested in various population measures including the significantly higher effective size of Africans compared to populations from other continents. One feature of this is the marked elevated heterozygosity across the genome and low LD [1], [2]. However both are with inherent limitations in reflecting the true extent of genetic variation within a locus or entire genome and its genealogy [2]. The first evidence to relate the *Homo sapiens sapiens* origin, exodus and dispersal out of an African birth place, was based on the mitochondrial control region variation in a global sample of 147 females [3]. This is the chronology suggested by classical protein markers [4], and subsequently consolidated by genetic data from whole mitochondrial genome sequence [5], Y chromosome [6] and autosomes [7], [8]. The advent of large scale sequencing made whole mitochondrial genome sequencing the method of choice for evolutionary studies, gradually replacing the control region sequencing which has extreme variation in substitution rate among sites, and the consequent parallel mutations [5], [9], [10]. Nevertheless, whole mitochondrial genome sequencing, though capable of providing reliable overall phylogeny based on averaged variation between sequences, its estimate of dates, population size and structure could still potentially be influenced by conflict in evolutionary rates between sites and the genetic noise of HVRI and HVRII excess mutations [11]. The small non-recombining genome of the mitochondria allows a single gene or sequence to answer specific evolutionary questions that would reflect patterns obtained from the whole sequencing [12]. Although studies addressing sequence variation in mtDNA coding region have suggested that natural selection has significantly shaped the course of human mtDNA evolution [13], [14], there is disagreement upon whether the distribution of specific human mtDNA haplogroups is due to an adaptation to different climates or if their distribution is a function of random drift assisted by purifying selection that eliminates non-synonymous changes [15]. The significance of a sequence displaying selective neutrality feature is its enhanced utility in estimating effective population size, one of the challenging and controversial undertakings in evolutionary genetics. Whole genome microsatellite or single nucleotide polymorphism data sets reflects features of selective neutrality in an averaged manner, as the intensities of selective signals vary widely between chromosomes and genomic regions. Despite a consensus on a large African effective population size as compared to populations from other continents, the extent of human genetic variation is still poorly understood particularly in east Africa. mtDNA is one of the most convenient tools in resolving questions of population size and genetic diversity given its extended haplotype structure, non-recombining nature and uni-parental inheritance. These features combined, apart from the non-recombining portion of the Y chromosome, are present nowhere else in the human genome and hence make mtDNA variation ideal for screening large data sets. Furthermore inference made from the mtDNA hyper-variable control region or one of its coding genes are found to be highly correlated with whole mitochondrial DNA sequencing thus providing an added value for identifying population gaps in resource limited settings. In the current study, we explore the level of genetic diversity among east Africans utilizing a genome wide set of microsatellites and MT-CO2 sequences to gain insights into the extent to which the east African gene pool has contributed to genetic variation at a global scale.

## Results

Table 1 shows the population parameters and selective neutrality test (Tajima's D) based on MT-CO2 variation of all continental groups, mean values and test of significance for the obtained values. Tajima's D (Table 1) scored negative values consistent with human expansion within and outside of Africa (or exchange of alleles between neighboring demes, see discussion below) with satisfactory statistical scores. The transition to transversion ratio of 2∶1 in our reported Single Nucleotide Polymorphisms (SNPs) is consistent with being at the root of the gene tree and with neutral evolution distance based analysis using *FST* for mtDNA sequences and *RST* for microsatellite data were carried out for subsequent MDS plotting and a population by population correlation comparison using Mantel Test. The result showed no correlation with a *P* value of 0.66 similar to comparison between mitochondria and Y chromosome variations reported earlier [16].

**Table 1:** Number of sequences sampled, population parameters and selective neutrality test (Tajima's D) based on MT-CO2 variation of all continental groups, mean values and test of significance for the obtained values.

	East Africa	Africa	Asia	Europe	America	Australia	Mean	s.d.
Sample size	172	53	20	45	20	18	54.66667	59.34869
S	39	13	3	7	3	5	11.66667	13.89484
Pi	1.90616	1.27576	0.38947	0.51111	0.55789	0.65359	0.88233	0.59014
Tajima's D	−2.12538	−1.64669	−1.44071	−1.83924	−0.90875	−1.74211	−1.61714	0.41402
P value	0.00000	0.02200	0.07800	0.00900	0.22200	0.01600	0.05783	0.08500

### Effective Population Size and Bayesian Skyline Plot (BSP) analysis

Given the intense debate between those advocating the use of a phylogenetic mutation rate calibrated by the divergence between humans and chimpanzees and those studying the mutation process on pedigrees, we attempt to calculate the *Ne* values using different loci and mutation rates to emphasize the relation of the *Ne* values to both parameters. The estimated *Ne* based on MT-CO2 in east and other parts of Africa and the rest of the world based on three different mutation rates of 9.2×10^−7^, 3×10^−6^ [17] and 3.15×10^−7^ [14] substitutions per site per generation of 25 years gave values that ranged between 6,195 at the lowest to 58,997 at the highest for east Africa (Table 2). *Ne* estimates based on microsatellites loci also using three different mutation rates [18], [19] still gave higher value of 26,734–1,782,277 for east Africans, though the fold increase in *Ne* was lower than that obtained by MT-CO2 (Table 2).

**Table 2:** Values of *Ne* calculated using a maximum likelihood estimate (MLE) of theta value generated by LAMARC version 2.1.8 for all continental populations and selected African groups.

Region	Population Name	MLE θ MT-CO2	*Ne*			MLE θ STRs	*Ne*		
			µ^a^ = 9.2×10^−7^	µ^b^ = 3×10^−6^	µ^c^ = 3.15×10^−7^		µ^d^ = 1.5×10^−4^	µ^e^ = 1.52×10^−3^	µ^f^ = 1×10^−2^
**All Eritrean populations**		0.012314	**6,692**	**2,052**	**19,546**	na	**na**	**na**	**na**
**Sudan**	**Nilotic**	0.012	**6,522**	**2,000**	**19,048**	475	**792,281**	**78,186**	**11,884**
**Sudan**	**Beja**	0.01185	**6,440**	**1,975**	**18,810**	883	**1,472,296**	**145,292**	**22,084**
**All Sudan populations**		0.026561	**14,435**	**4,427**	**42,160**	621	**1,034,192**	**102,058**	**15,513**
**Africa**	**East Africa**	0.037168	**20,200**	**6,195**	**58,997**	1,069	**1,782,277**	**175,883**	**26,734**
**Africa**	**Southern Africa**	0.006219	**3,380**	**1,037**	**9,871**	688	**1,146,588**	**113,150**	**17,199**
**Africa**	**North Africa**	0.00148	**804**	**247**	**2,349**	na	**na**	**na**	**na**
**Africa**	**The rest of Africa**	0.009299	**5,054**	**1,550**	**14,760**	750	**1,249,727**	**123,328**	**18,746**
**Americas**	**Americas**	0.001704	**926**	**284**	**2,705**	529	**881,557**	**86,996**	**13,223**
**Asia**	**Asia**	0.001825	**992**	**304**	**2,897**	871	**1,451,471**	**143,237**	**21,772**
**Australia**	**Australia**	0.003517	**1,911**	**586**	**5,583**	239	**397,981**	**39,274**	**5,970**
**Europe**	**Europe**	0.003853	**2,094**	**642**	**6,116**	607	**1,011,304**	**99,800**	**15,170**
**Africa**	**whole Africa**	0.03855	**20,951**	**6,425**	**61,190**	1059.523	1,765,872	174,264	**26,488**
	**whole world**	0.03764	**20,457**	**6,273**	**59,746**	834.3477	**1,390,580**	**137,228**	**20,859**

a Mutation rate calculated from observed variations in MT-CO2 this work, ^b^Jazin *et al*., 1998 [17], ^c^Mishmar *et al*., 2003 [14], ^d^Zhivotosksy *et al*., 2000 [56], ^e^Erikson and Monica 2011 [19], ^f^for comparison.

Theta values are based on analysis of mtDNA sequences and microsatellite alleles.

**Figure 1:**
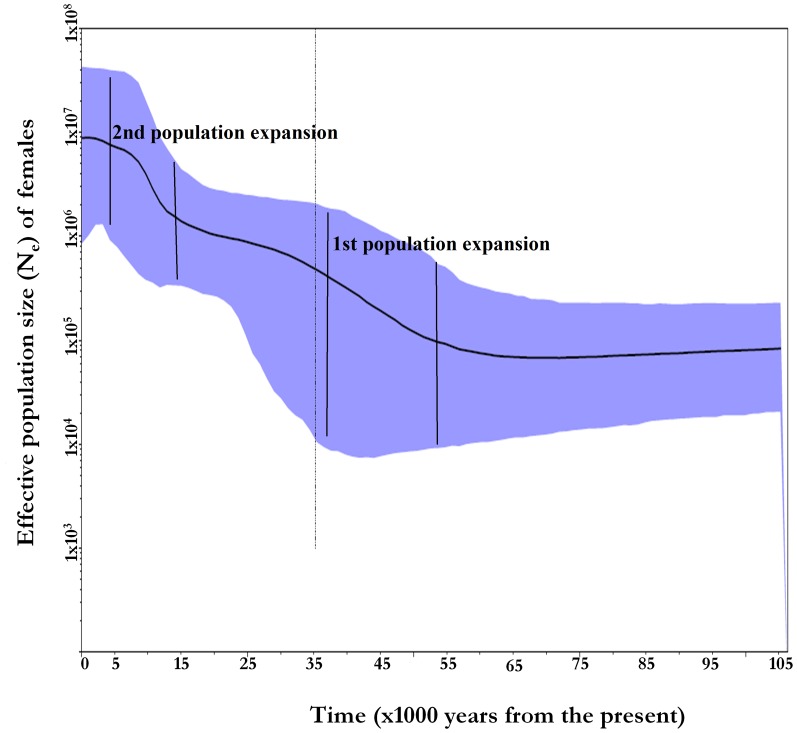
Bayesian Skyline Plot (BSP). BSP based on 543 base pairs of mitochondrial DNA MT-CO2 region. The graph was constructed merging all populations as global population. The plot displays changes in world female effective population size (*Nef*) through time, a 25 year generation time, and a 9.2×10^−7^ sub/site/generation mutation rate. Present day is on the left on the x-axis. An increase in world female population observed at around 50, 000YBP and around 10, 000–20,000YBP (the periods are highlighted).

As *Ne* can also differ when taken in different points in time; we did a Bayesian analysis enabling the growth rate option in the program LAMARC and found effective population growth at different generations in the past of the archaic *Ne* (Table 3). The expansion was first observed around 50, 000YBP with a doubling in the current effective population size observed at around 15, 000YBP. In all cases East Africa maintained a higher effective size of at least two to six times that of other populations including other Africans. The BSP (Figure 1, and supplementary Figures S5A–C) analysis also confirmed the results from LAMARC with the two major expansions starting around 50, 000YBP after a long steady decline of population size since coalescence time and another around 15, 000YBP, albeit with higher estimates of archaic and current *Ne*. Bayesian Skyline plots of individual populations (supplementary Figures S5A–C) depict varied population histories including time of coalescence and expansion and conform to large extent to mismatch plots of single groups.

**Table 3:** Current effective population sizes for all continental populations and selected African groups were calculated from the theta value generated by LAMARC version 2.1.8 by employing a Bayesian approach.

Bayesian *Ne* and growth rate using mtDNA sequences										
Populations	Growth rate	Theta current	*Ne* current (µ = 9.2x^−7^)	*Ne* current (µ = 3.15x^−7^)	*Ne* archaic (800) (µ = 9.2x^−7^)	Ne archaic (800) (µ = 3.15x^−7^)	*Ne* archaic (2000) (µ = 9.2x^−7^)	*Ne* archaic (2000) (µ = 3.15x^−7^)	*Ne* archaic (4000) (µ = 9.2x^−7^)	*Ne* archaic (4000) (µ = 3.15x^−7^)
Eritrea	933.5889	0.020262	**11,012**	**32,162**	5,539	16,178	1,976	5,772	355	1,036
Australia	647.9034	0.011588	**6,298**	**18,394**	3,909	11,418	1,912	5,584	580	1,695
North Africa	945.5722	0.010429	**5,668**	**16,554**	2,826	8,254	995	2,906	175	510
Americas	936.4635	0.010608	**5,765**	**16,838**	2,894	8,452	1,029	3,006	184	537
East Africa	976.2668	0.092493	**50,268**	**146,814**	24,504	71,567	8,340	24,357	1,384	4,041
Rest of Africa	920.1735	0.015341	**8,338**	**24,351**	4,236	12,371	1,534	4,479	282	824
Europe	942.6523	0.010615	**5,769**	**16,849**	2,883	8,419	1,018	2,974	180	525
Beja	902.324	0.033477	**18,194**	**53,138**	9,365	27,352	3,458	10,101	657	1,920
Nilotics	926.3229	0.030164	**16,393**	**47,879**	8,290	24,214	2,982	8,708	542	1,584
Whole Sudan	951.5374	0.084104	**45,709**	**133,498**	22,691	66,271	7,936	23,179	1,378	4,025
Asia	937.3591	0.010732	**5,833**	**17,035**	2,926	8,545	1,039	3,036	185	541
Whole Africa	976.947	0.093893	**51,029**	**149,037**	24,862	72,614	8,455	24,695	1,401	4,092
Whole World	979.251	0.089875	**48,845**	**142,659**	23,758	69,389	8,059	23,538	1,330	3,884

Archaic theta was calculated using the formula: theta (at time t) = theta (now)EXP(−gt).

*Ne* is calculated at different generations (in bracket) in the past using mutation rate of 9.2×10^−7^ substitutions/site/generation.

The archaic *Ne* at different time (in generations) interval in the past was calculated subsequently after the archaic theta was calculated from the current theta using a formula: theta (t) = theta (now)*EXP (growth rate*t*mutation rate).

### Effective Size versus Census Size

**Figure 2:**
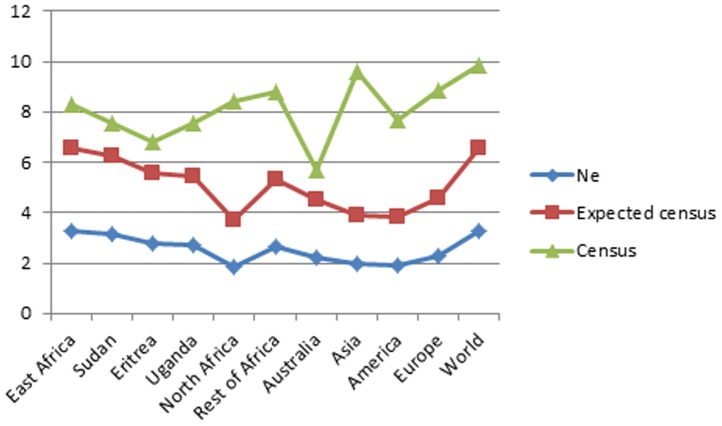
Line Plot. Line plot of the effective size (lower), expected census size (middle line) and actual census size (upper) of the continental populations and some east African populations. Each point represents the log of actual values of the census, expected census and effective size of these populations. The expected census is obtained by calculating the current *Ne* by 10.

A line graph (Figure 2) plots the log values of both *Ne* based on MT-CO2 and real and expected census size of east Africans, the rest of Africans and other continents [20]. The figure depicts an increased effective size in Africa and near the root in east Africa, relative to census size, while the trend is reversed in the out of Africa groups with exception of Australians among which considerable mtDNA diversity seems to have been maintained. Fisher's exact test performed to assess the difference between the observed and expected census values in Aboriginal Australians did not reject the null hypothesis for association (one tailed *P* = 0.06) between the *Ne* and expected census size, usually taken to be ten times the effective size [21], [22].

### Mutations and Haplotypes Frequencies in the MT-CO2

The sheer number of haplotypes, a basic measurement of genetic diversity, is also taken as an indication of *Ne*. As mitochondria are non-recombining the number of mutations and haplotypes is quite correlated. In the MT-CO2 sequence 68 haplotypes were estimated using Arlequin ver3.11 and assigned numbers from 1 to 68. Haplotype relative and absolute frequencies in the studied populations were also calculated. Strikingly, of the total 68 haplotypes, 43 occurred solely in east Africa (Table S1) of which 25 were in Sudanese, 9 in Eritreans and 5 in Ugandans and one Kenyan. The rest of the haplotypes were derived from or included east Africans with exception of 13 haplotypes, 4 in Africa 2 in Australia, 3 in Europe 1 in Arabia 1 America/Africa and 1 Europe/Africa. Of the 42 haplotype defining mutations (Table S2) in Sudanese and Eritreans 11 (26.2%) were non-synonymous (replacements) occurring in trans-membrane domain of COII protein while 31 (73.8%) were synonymous with transitions representing the majority of the mutations. Out of the 42 mutations (Table S2), 31 were previously reported in the literature and 11 were novel. All mutations in Ugandan MT-CO2 samples are synonymous and reported at http://dspace.nwu.ac.za/handle/10394/4221). All published haplogroups associated with the mutations are indicated in Table S2.

### Phylogenetic Inference: Median-Joining (MJ) Network and Neighbor Joining (NJ) Tree based on MT-CO2 Sequences

**Figure 3:**
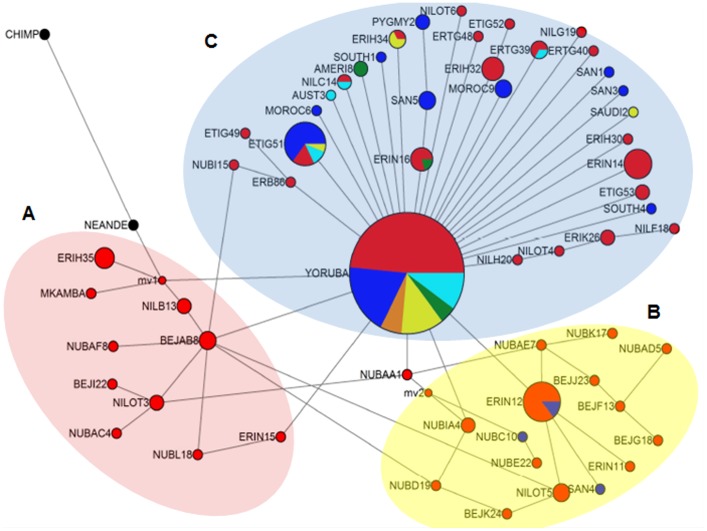
Median joining (MJ) Network tree. MJ tree based on MT-CO2 gene sequences of world populations. Black points indicate, root; Blue, East Africans; Red, Africans; Orange, Australians; Green, Asians; Pink, Americans; Purple, Europeans. Branch lengths are not representative of evolutionary distance. The background colors outline possible demographic events in east Africa, and early episodes of evolution possibly towards the Rift Valley.

The most ancestral haplotypes as judged by Neanderthal and Chimpanzee was a group of sequences that diverged early in human history forming a cluster denoted as (A) that includes 1 individual sequence from the Rift Valley Kenya (Mkamba), in addition to Eritreans, Ugandans and Sudanese; another branch differentiate encompassing solely east African, predominantly Eritreans, Ugandans and Sudanese and denoted as cluster (B). The third cluster (C), includes members of almost all world populations particularly non-Africans who share a major haplotype that seems to have originated within an east African gene pool (Table S1 and Figure 3). The starry shape of the major haplotype (Figure 3) and values of Tajima's D indicate that the ancestral population has undergone at least one episode of expansion prior to the dispersal that led to the migration of our species out of Africa.

This was also manifested in a neighbor-joining (NJ) tree of all MT-CO2 sequences (Figure S1). In agreement with the network (Figure 3) the tree shows the root to be unequivocally occupied by east Africans (Red dots) and the dispersal of their sequences along the tree and at its tip particularly individuals from Eritrea and Ethiopia consistent with what had been found when other east African populations were analyzed [10].

Given the uncertainty in the value of divergence between Humans and Chimpanzees reported in the literature, and the wide range of dates for the divergence of Humans from Neanderthal and Humans MRCA and the differentiation of major mtDNA and Y chromosome haplogroups [14], [23], [24]. We adopted the lower threshold of 0.5 million for divergence of our species from Neanderthal and its most recent common ancestor to be ∼100,000 years and the divergence of the major haplotype in Africa to be ∼50, 000YBP [25], less than reported elsewhere [14], [26].

### Multidimensional Scaling of Microsatellite and MT-CO2 Data

**Figure 4:**
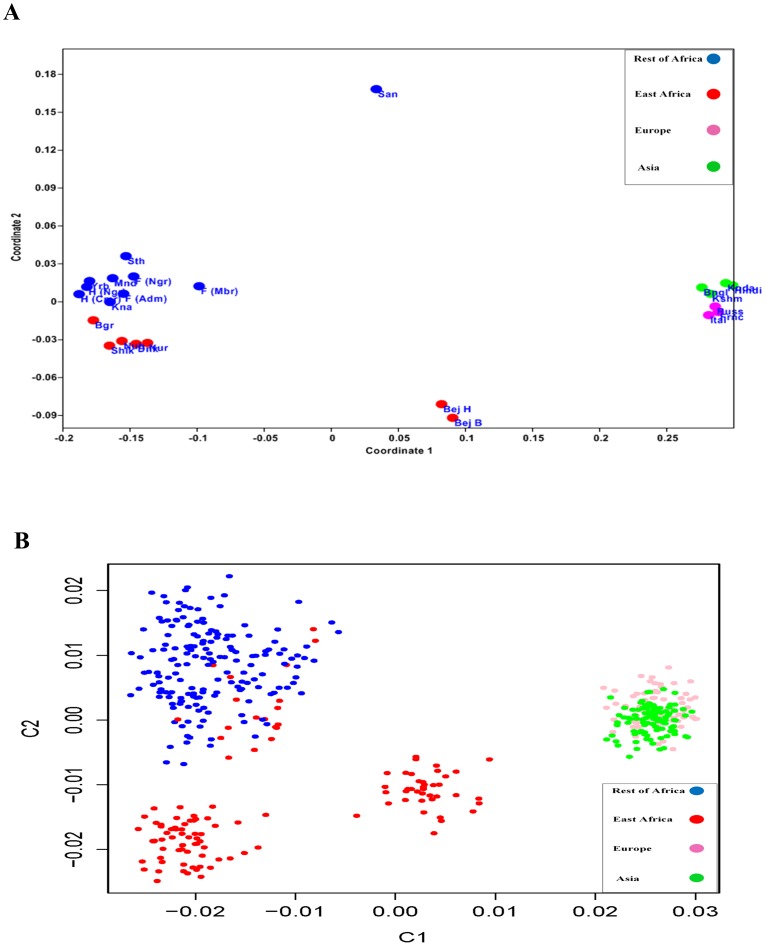
Multidimensional Scaling Plot (MDS). **A**. First and second coordinates of an MDS plot of 848 Microsatellite Marshfield data set across the human genome for 24 populations from Africa, Asia and Europe. MDS plot was constructed from pairwise differences *FST* generated by Arlequin program (Table S3). **B**. First and second coordinates of an MDS plot of 848 Microsatellite loci, across the human genome in 469 individuals from 24 populations from Africa, Asia and Europe. MDS uses pairwise IBS data based on the 848 loci generated by PLINK software and plotted using R version 2.15.0. East Africans cluster to the left of the plot, while Beja (red cluster in the middle), assumes intermediate position.

The difference in population size and divergent history of the world populations based on Marshfield genome wide microsatellite data set are also depicted in a multi-dimensional scaling (MDS) plots. The first plot employs an *FST* based distance matrix (Figure 4A) while the other is a PLINK calculated matrix based on probability of identity by state (IBS) for individual samples (Figure 4B). Both MDS plots discriminated between Africans and non-Africans in their first coordinate, although in the IBS plot where the variance measure is more pronounced the drift effect and low *Ne* is prominent. In the *FST* based MDS the 2^nd^ coordinate differentiates between Sudanese and the rest of Africa (except San), and between Asians and Europeans. Interestingly Beja population from Sudan maintains an intermediate position between Africans and non-Africans in both plots. The 3^rd^ and 4^th^ coordinates of the IBS plot appears to display subsequent demographic events where Africans particularly East Africans contributed independently to Europeans and Asian ancestry (Figures S2 and S3).

An MDS of the MT-CO2 data set (Figure S4) which includes additional populations to the microsatellite set, maintains elements of the clustering witnessed in other types of analysis in the present manuscript and literature, for example the association and central contribution of East African populations particularly Nilo-Saharan to early demographic events in Human history suggested by the first coordinate; the relationship between Nuba and Nubians and Moroccans and Ethiopians as shown in the second coordinate which appears to express elements of the out of Africa episode (s).

## Discussion

The tenet of the human population genetic structure is best viewed in the light of the inherent diversity within the human genome. Humans are believed to have expanded from small founder demes somewhere in eastern Africa during the past 75,000-100,000YBP [27] even though debatable. However, much need still exists to unravel the forces behind this variation; as well as explain and date the demographic history [28]. A definitive answer to questions on population size, and past evolutionary dynamics of African human populations has been hampered, hitherto, by substantial gaps in sequence information from African population even though it is generally expected that it is the area with the most substantial variation existing unlike anywhere else. In particular, east Africa has been suggested by the shreds of existing data to be pivotal to recent and early human evolution [10], [29]. In the present study we provide evidence of a significantly larger east African population size, based on various genome markers, most prominently sequence data from the mitochondrial MT-CO2, and autosomal microsatellite genetic variation.

Historically, the MT-CO2 gene has been employed in species and phylogenetic inference for the various merits mentioned earlier, and inference made from such sequence information are found to be highly correlated with large data sets and whole genome analysis [5]. Of the few studies done to evaluate the validity of using single conserved mtDNA gene as an evolutionary tool, MT-CO2 was used to estimate divergence time between modern humans and chimpanzee depending on class 1 mutations and maximum likelihood analysis [30]. Its conserved functional nature gives values of selective neutrality attested by a general excess of transitions and other relevant genetic parameters. Likewise, the bulk of mutations that underlies this striking diversity in our data set exhibits similarly features of selective neutrality, with excess of transitions and negative values from Tajima's D test, hence are unlikely to be explained by a scenario of a selective sweep including the major haplotypes cluster that defines expansion within Africa and a subsequent out-of-Africa exodus event. The confinement of non-synonymous mutations to east Africans (mainly Sudanese) is consistent with a position at the base of the human evolutionary tree. Based on nested contingency analysis of MT-CO2 sequences in humans and primates, Templeton [31] interpreted on biological grounds that trans-membrane replacement mutations seems to behave in a neutral fashion. It has been argued recently that even in a scenario of natural selection, the observed differences in fitness components may likely be evolutionarily neutral and small selective differences in life histories will be hard to measure, hence the effects of random drift will be amplified in natural populations by the large variances among individuals [32].

According to the current data East Africa is home to nearly 2/3 of the world genetic diversity independent of sampling effect. Similar figure have been suggested for sub-Saharan Africa populations [1]. The antiquity of the east African gene pool could be viewed not only from the perspective of the amount of genetic diversity endowed within it but also by signals of uni-modal distribution in their mitochondrial DNA (Hassan *et al*., unpublished) usually taken as an indication of populations that have passed through “recent” demographic expansion [33], although in this case, may in fact be considered a sign of extended shared history of *in situ* evolution where alleles are exchanged between neighboring demes [34].

We compare and contrast the MT-CO2 sequence data to analysis of 848 microsatellites of the Marshfield dataset partly published by Tishkoff *et al*. [8] and find convincing correlation and a persistent pattern where Africans and particularly east Africans show greater variance in both distance and Bayesian based analysis. Although such large population size is expected to reflect on the spectrum of SNPs and haplotype structure in the human genome, an in-depth investigation is not currently feasible in the absence of major sequencing efforts in east Africa. However, an impression based on available sequences of X-chromosome and candidate gene analyses indicate that this ratio does persist across data for east Africans (unpublished data from the MalariaGen consortium and exome sequencing from our own group).

Our estimate of the population size is more in agreement with LD and coalescent dates as in Tenesa *et al*. [2]. Difference in estimate of archaic population size between studies in the range of 1000–10000 [2], [24], [35] has been attributed to *Ne* being estimated over different points in time [2]. Emphasis should be laid as well on the evolutionary rate of the genetic loci used in the analysis. The 10,000 figure suggested by Takahata *et al*. [24] was based on MHC polymorphisms, a locus with one of the highest evolutionary rates in the human genome. We base our estimates on two classes of loci a mitochondrial gene with an intermediate evolutionary rate and, evidence of selective neutrality (MT-CO2), and genome wide microsatellites, a fast evolving high mutation rate type of loci similar to HLA. Although larger *Ne* of east Africans was consistently maintained, microsatellites produced values in the upper extremities of *Ne*. The size of difference for microsatellites was much narrower possibly due to the fact that higher mutation rates may lead to convergence, in addition to admixture, both elements that are known to affect the resolution of these markers in coalescence estimates. This should also be considered against arguments that estimates based on mitochondrial genome, tend to be biased towards low inbreeding effective size because of unique features pertaining to the mitochondrial genome [31]. Such disparities in the estimates of effective population size when using different genes and molecular markers [2], [24], [35] including the mitochondrial genome [36] may also account for discrepancies in the dating of the age of demographic events based on mitochondrial genome or the human genome in general. The other factor that seems to influence the outcome of the analysis most is the effective size itself or the sheer number of variants in the target genome or loci. The report of larger number of mutations in the MT-CO2 gene here, has led to different estimate of a mutation rate by a factor of 10. Interestingly there seems to be a trend for bringing the dates of coalescence more close to the present time and the population size smaller [2], [37], [38]. Based on Bayesian analysis of three previously analyzed nuclear loci sequences [38] reported *Ne* of 7,000 for long term effective size, in tally with our figures. Interestingly the two expansions suggested by the BSP coincide with major putative demographic events: one the critical biological changes that might have taken place around 50, 000YBP subsequently leading to the out of Africa expansion; and the other is advent of pastoralism and agriculture believed to have occurred around 10, 000–20, 000YBP, as the latter date was also attested by Y-chromosome [39], autosomal and mitochondrial HVRI re-sequencing analyses [40]. These two demographic events are also corroborated by whole mitochondrial sequence analysis [41].

Previous anthropological and genetic evidence have suggested that long-term *Ne* has been about three times larger in African populations than in non-Africans [1], [35], [42]. Simulations studies of Africa, Europe and Asia, suggest that the African effective size is still the largest of the three regions and is probably at least as great as the sum of the Asian and European effective sizes. Our data set, however, indicate that east African population size alone may be ten times larger than that of Europeans and Asians and three times that of other populations including Africans. In fact of 68 MT-CO2 haplotypes only 14 were unique to populations other than east Africans. Re-sequencing of ∼8 Mb in 20 independent non-coding autosomal region of different continental populations also pointed to a five times reduction of effective population size within the out of Africa migrants relative to an ancestral African population size [43]. The main point to be stressed here is that given the observation of east Africa consistently displaying a relatively higher long term population size, we conclude that human population size might be larger than anticipated.

A relatively low effective size and the drift like nature of the event characterizing the group that made the exodus out of Africa, is manifested in the fact that it originates in a spectacularly large female source in east Africa. This is in concordance with previous data including X chromosome. In a recent report by Keinan *et al*. [7] the authors conclude that a sex-biased process that reduced the female effective population size, or an episode of natural selection that unusually affected chromosome X, was associated with the founding of non-African populations.

The complex relationship between the census and effective size is influenced by both genetic drift and inbreeding. The drift nature of the out of Africa event and the subsequent demographic expansion is portrayed in the relationship between the census and effective size in east Africa and the rest of the world particularly Asia and Europe. Both drift and inbreeding are unlikely to have influenced east Africans in a substantial way due to the limited geographic range where those groups evolved and in-situ evolution of its population.

Fluctuation in population size might be a mark of the out of Africa group because migration and challenges of adapting to new environments subject the population to both influences of drift and inbreeding. Cases of low census size and a larger inbreeding effective size are known in mammalian populations and attributed to recent population reductions [44]. Although the difference between the current and expected census for Australians was not statistically significant it still indicates an interesting feature of this isolated group. It is not clear why Australia was colonized with a higher population size than the populations that colonized other regions. Henn *et al*., [45] contemplated this in the light of lineage specific acceleration. Our findings, however, indicate that the population of Australia may have maintained a legacy of high *Ne* originally carried by the ancestral group that left Africa and seen in the number of haplotypes that survived in their gene pool. This may suggest that both census and effective size of the group that made it to Australia was large enough to counteract the effect of drift and permit survival of relics of these original haplotypes.

It is not only genetic data that lends support to an east African origin of humans but the unparalleled ethnic and linguistic diversity that remains one of the highest worldwide. Interestingly the two most ancestral sequences in the NJ tree figure refer to Nubian individuals. Nubia is currently identified with one of the most ancient human settlements, the Say culture. Recently, a related compound associated with a lithic middle Stone Age industry was discovered in Dhofar Oman and taken as an evidence of human migration out of Africa through an Arabian route [46].

Overall, the various genetic markers used in the current analysis support the observation of human effective population size larger than previously estimated, and emphasize the importance of sampling populations of putative deep ancestry.

## Materials and Methods

### Ethical statement

Ethical approval has been obtained from regulatory bodies in Sudan (Ethics Review committee (ERC) Institute of Endemic Diseases, University of Khartoum: http://www.healthresearchweb.org/br/sudan/ethics; Reference: 28/10/03etiend) and Eritrea (The Ethical Review Board of Eritrea Institute of Technology, Eritrea; Reference: June 21, 2011) for the sample sets analyzed in the Khartoum laboratory. Other data has been obtained from online sources. All past and present research adopts good practices in ethics and local and international guidelines for genetic research. This includes obtaining of written informed consents from all participants, keeping the data anonymity from the beginning and communicating research results to populations concerned.

### Data sets, Sequences and alignment

Data sets used in this study include genotype information on 848 nuclear microsatellites obtained from individuals of various world populations including Sudanese, Ethiopians and Kenyans available at http://www.sciencemag.org/content/324/5930/1035/suppl/DC1 and MT-CO2 DNA sequences comprising 23 Ugandans obtained from http://dspace.nwu.ac.za/handle/10394/4221), 75 other Africans, 20 Asians, 18 Australians, 45 Europeans and 20 Americans making a total of 180 sequences obtained from public mtDB database (http://www.mtdb.igp.uu.se/) in addition to 81 Eritreans and 46 Sudanese sampled and genotyped following appropriate ethical consent and documentations. PCR amplification of MT-CO2 gene for the Eritreans and Sudanese samples was carried out at the Institute of Endemic Diseases, University of Khartoum and outsourced for commercial sequencing at BGI Hong Kong, China. MT-CO2 sequences were identified by blasting against Yoruba reference sequence (accession number AF347015) and sequences with mutations were deposited at GenBank with accession number KC753688-KC753760.

A total of 330 MT-CO2 sequences were aligned using the software program BioEdit [47]. Yoruba reference sequence (GenBank accession number AF347015) was used as a reference sequence for the alignment. Polymorphic positions were visually noted and scored. For Eritrean and Sudanese populations, mutations were confirmed in forward and reverse electropherograms. Synonymous, non-synanymous and novel mutations are indicated in Table S1. Arlequin ver3.11 [48] was used to calculate the relative and absolute haplotype frequencies among study group sequences in addition to the shared haplotypes (Table S2).

### Statistical analyses

Arlequin suite ver3.11 [48] was used to provide information on genetic and demographic features of the studied populations. Haplotypes frequencies, pairwise *FSTs*, in addition Tajima's D test to check for selective neutrality were calculated for the studied populations. Fisher's exact test was used to test the probability of association between the expected census size being ten times the effective size and observed census size for current population of aboriginal Australians. The correlation between the two markers was evaluated by Mantel Test within Arlequin program.

### Populations' structures and affinities

MDS (Figure 4A) was plotted from *FST* intermediate values (Table S3), generated from Arlequin version 3.1 using Marshfield microsatellite allele frequency data, and employing cluster analysis option within the program PAST (Paleontological Statistical Tool program [49]. Versus the distance approach we also employ a Bayesian approach using a device suggested by Cavalli-Sforza and implemented by Reich and Patterson [50] to transform the microsatellite data into identity by state (IBS) Each allele is considered as a separate marker and reported as having one copy, two copies or no copies. The data thus comes to resemble bi-allelic data. The data was pruned by removing all markers with more than 10% missing data, then analyzed and plotted (Figure 4B) using PLINK program [51] and graph drawn with R version 2.15.0.

### Phylogenetic trees and network

Phylogenetic tree was constructed for the MT-CO2 sequences using Neighbor-Joining (NJ) method. The NJ tree was calculated in MEGA5 [52]. Both Chimpanzee (GenBank accession No D38113) and Neanderthal (EMBL accession number AM948965) sequences were used as out groups. The aligned and refined MT-CO2 sequences were used to construct a Median Joining (MJ) Network using Network 4.6.1.1. (http://www.fluxus-engineering.com) as outlined by Bandelt *et al*. [53]. Dating was carried out using a coalescent based analysis and SkyLine Plot Bayesian inference, with options available in the programs Network [53] and BEAST [54]. The mutation rate was estimated at 0.5–1.0 nucleotide/site/100.000 years referring to published data on dates of divergence between humans and chimpanzee.

### Effective population size

The effective population size (*Ne*) was calculated using an MLE (maximum likelihood estimate) of theta value generated by LAMARC version 2.1.8 [55]. The method uses a coalescence approach to estimate population parameters by sampling random genealogies of sequences (or alleles) to calculate the parameters and Metropolis Monte Carlo sampling technique to concentrate the sampling in regions which contribute to the final result. The log value of the *Ne* and the census size was used to plot the chart. Current and archaic *Ne* values (Table 3) were also calculated from current and archaic theta values after generating growth rate and Bayesian estimation of current theta using LAMARC program. Archaic theta was calculated using the formula theta (t) = theta (now)EXP(−gtμ).

### Bayesian Skyline Plot analysis

In order to assess past changes in female effective population size (*Ne*) further, we used the BEAST v1.8.0 [54] package to analyze timing and magnitude of past changes in population size. We analyzed the datasets under the HKY model putting the clock model as relaxed uncorrelated clock with the mean mutation rate of 9.2×10^−7^ substitutions per site per generation as used in LAMARC. We used a Bayesian Skyline coalescent tree prior with 10 groups under a piecewise-constant model. The analysis was run for 10 million generations with parameters logged every 1000 generations, and Tracer 1.6 (http://tree.bio.ed.ac.uk/software/tracer/) was used to inspect chain convergence and conduct the skyline reconstruction (Figure 1). BSP analysis (supplementary Figures S5A-C) was also conducted for each individual populations for comparison purpose.

## Supporting Information

Figure S1 **Neighbor joining (NJ).** NJ tree of the world populations based on MT-CO2 sequences. The evolutionary relationship of 171 sequences and evolutionary history was inferred using the Neighbor-Joining method. The optimal tree with the sum of branch length = 0.20401570 is shown. The evolutionary distances were computed using the Maximum Composite Likelihood method and are in the units of the number of base substitutions per site. Codon positions included were 1st+2nd+3rd+Noncoding. All positions containing gaps and missing data were eliminated from the dataset. There were a total of 543 positions in the final dataset. Phylogenetic analyses were conducted in MEGA4. Red dots: east Africa, Blue: Africa, Green: Asia, Yellow: Australia, Pink: Europe and gray: America.
(TIF)

Figure S2 **Multidimensional Scaling Plot (MDS).** The 2nd and 3rd coordinates of an MDS plot of 848 nuclear microsatellite loci from 469 individuals of 24 world populations. MDS uses pairwise IBS data based on the 848 loci generated by PLINK software and plotted using R version 2.15.0. The figure, besides a separate clustering of east Africans, indicates the substantial contribution of Africans and east Africans to the founding of populations of Europe and Asia.
(TIF)

Figure S3 **Multidimensional Scaling Plot (MDS).** The 3rd and 4th coordinates of an MDS plot of 848 Microsatellite loci, across the human genome in 469 individuals from 24 populations from Africa, Asia and Europe. MDS uses pairwise IBS data based on the 848 loci generated by PLINK software and plotted using R version 2.15.0. The central position of east Africans and some other Africans emphasizes the founding role of east African gene pool and the disparate alignment on coordinates along which the world populations were founded including populations of Aftica aligning along the 4th dimension.
(TIF)

Figure S4 **Multidimensional Scaling Plot (MDS).** First and second coordinates of an MDS plot based on MT-CO2 data set constructed from pairwise differences *FST* generated by Arlequin v3.11. Population code as follows: Nara: Nar, Kunama (Kun), Hidarb (Hid), Afar (Afa), Saho (Sah), Bilen (Bil), Tigre (Tgr), Tigrigna (Tig), Rashaida (Rsh), Nilotics (Nil), Beja (Bej), Ethiopians(Eth), Egyptians (Egy), Moroccans (Mor), Southern Africans (Sth), Pygmy (Pyg), Saudi Arabia (Sdi), Asia (Asi), Europe (Eur), Native Americans (NA), Australians (Ast), Nubians (Nub), Nuba (Nba)
(TIF)

Figure S5 **Bayesian Skyline Plots (BSP).** BSP for individual population to clarify the demographic events each populations. A. Global populations, B. Eritrean populations and C. Sudanese populations.
(TIF)

Table S1 **Synonymous, non-synonymous and novel mutations identified against Yoruba reference sequence from Sudanese and Eritrean MT-CO2 sequences.** The Ugandan mutations are previously reported at http://dspace.nwu.ac.za/handle/10394/4221).
(XLSX)

Table S2 **Frequencies of 68 MT-CO2 haplotypes resolved within continental populations, the haplotype Number 1 include 164 individuals from all world populations.** The rest, shared or unique haplotypes shows the name of the group possessing the haplotype.
(PPTX)

Table S3 ***FST*** **matrix containing intermediate values generated from Arlequin version 3.11 using Marshfield microsatellite data set.**
(XLSX)
