# Supplementary figures and images for: The Episode of Genetic Drift Defining the Migration of Humans out of Africa Is Derived from a Large East African Population Size
Source: PLoS One. 2014 May 20;9(5):e97674. doi: 10.1371/journal.pone.0097674 (PMC4028218; doi:10.1371/journal.pone.0097674)

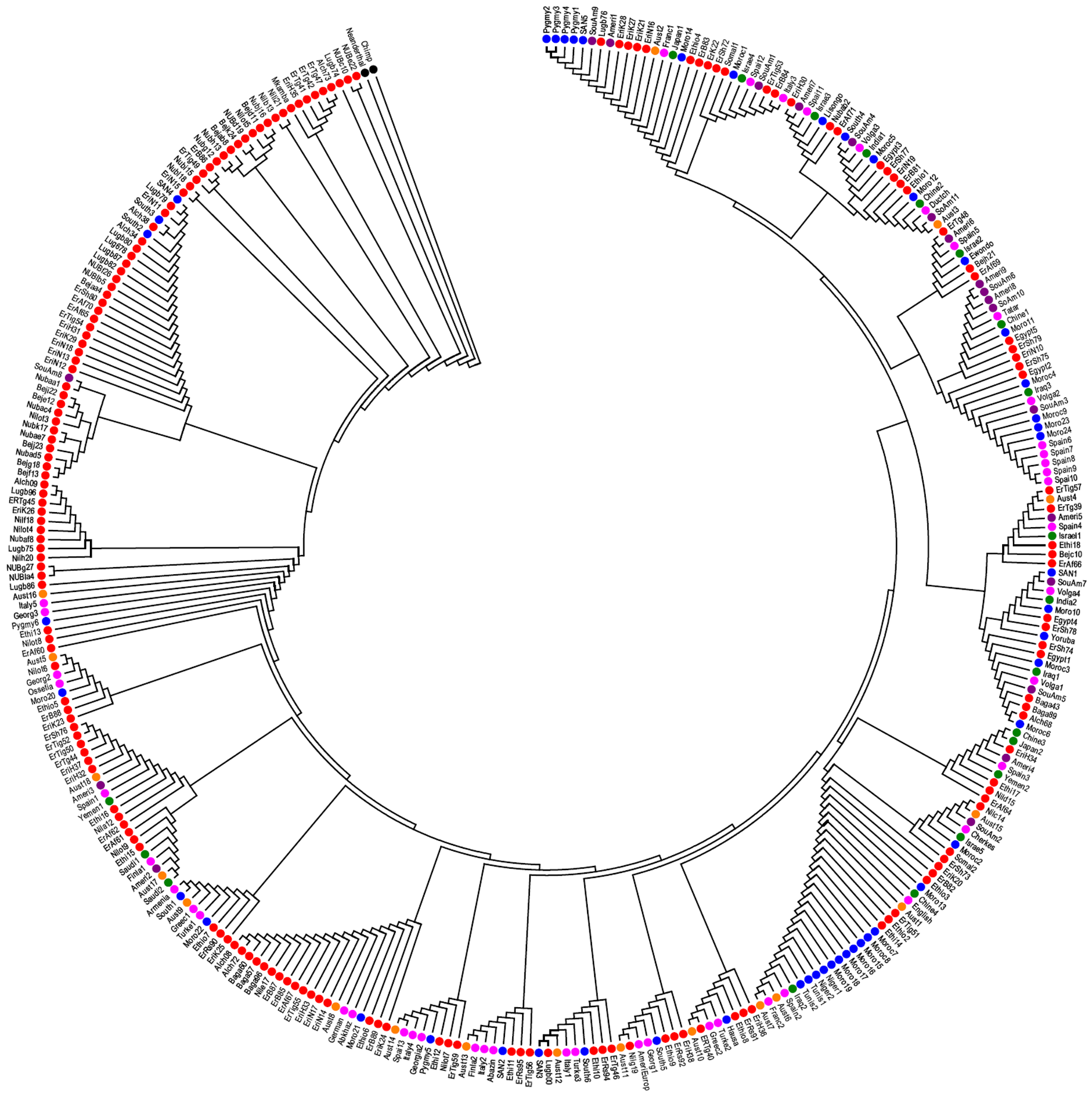

Supplement: Figure S1 — Neighbor joining (NJ). NJ tree of the world populations based on MT-CO2 sequences. The evolutionary relationship of 171 sequences and evolutionary history was inferred using the Neighbor-Joining method. The optimal tree with the sum of branch length = 0.20401570 is shown. The evolutionary distances were computed using the Maximum Composite Likelihood method and are in the units of the number of base substitutions per site. Codon positions included were 1st+2nd+3rd+Noncoding. All positions containing gaps and missing data were eliminated from the dataset. There were a total of 543 positions in the final dataset. Phylogenetic analyses were conducted in MEGA4. Red dots: east Africa, Blue: Africa, Green: Asia, Yellow: Australia, Pink: Europe and gray: America. (TIF) [file pone.0097674.s001.tif]

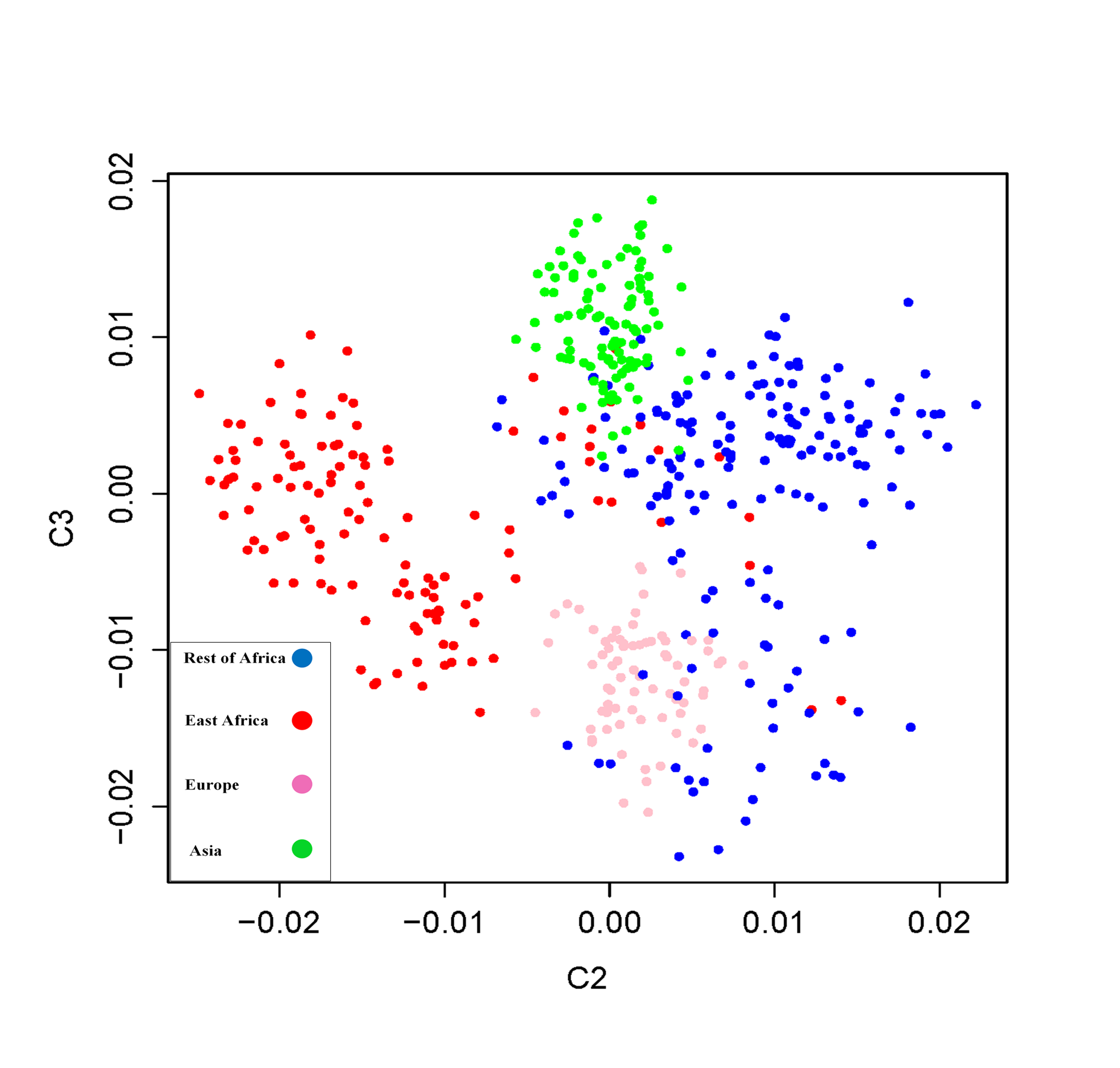

Supplement: Figure S2 — Multidimensional Scaling Plot (MDS). The 2nd and 3rd coordinates of an MDS plot of 848 nuclear microsatellite loci from 469 individuals of 24 world populations. MDS uses pairwise IBS data based on the 848 loci generated by PLINK software and plotted using R version 2.15.0. The figure, besides a separate clustering of east Africans, indicates the substantial contribution of Africans and east Africans to the founding of populations of Europe and Asia. (TIF) [file pone.0097674.s002.tif]

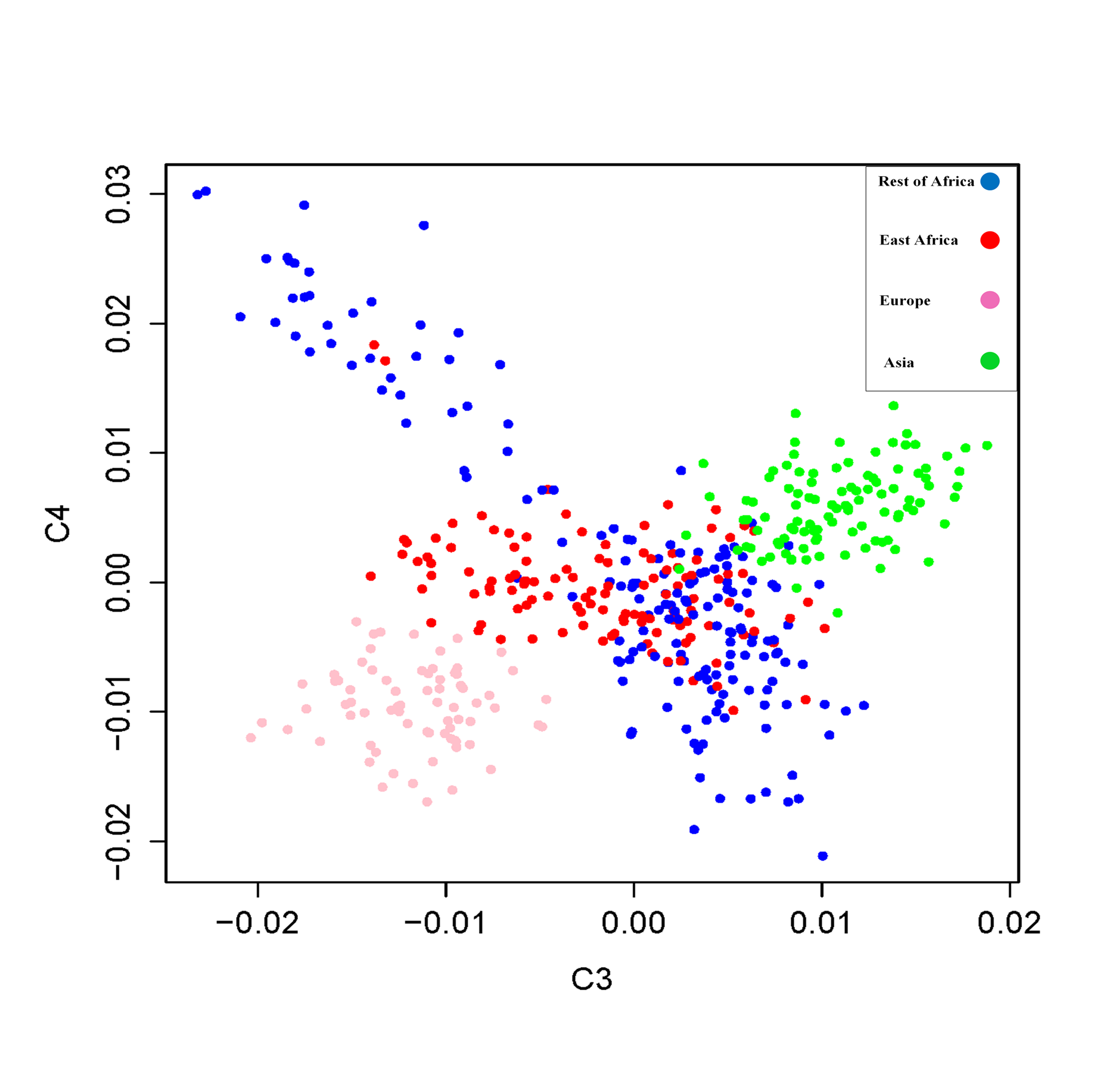

Supplement: Figure S3 — Multidimensional Scaling Plot (MDS). The 3rd and 4th coordinates of an MDS plot of 848 Microsatellite loci, across the human genome in 469 individuals from 24 populations from Africa, Asia and Europe. MDS uses pairwise IBS data based on the 848 loci generated by PLINK software and plotted using R version 2.15.0. The central position of east Africans and some other Africans emphasizes the founding role of east African gene pool and the disparate alignment on coordinates along which the world populations were founded including populations of Aftica aligning along the 4th dimension. (TIF) [file pone.0097674.s003.tif]

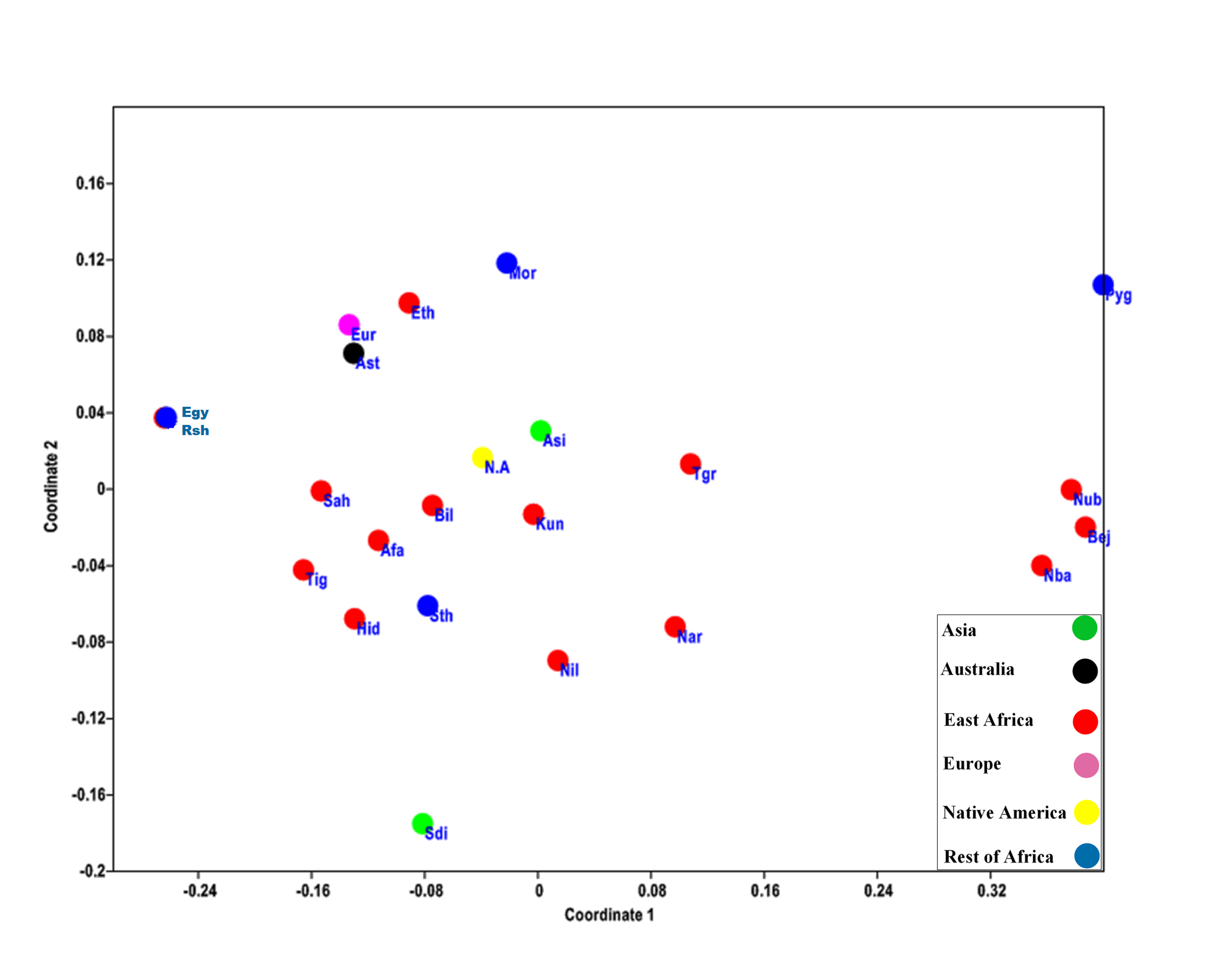

Supplement: Figure S4 — Multidimensional Scaling Plot (MDS). First and second coordinates of an MDS plot based on MT-CO2 data set constructed from pairwise differences FST generated by Arlequin v3.11. Population code as follows: Nara: Nar, Kunama (Kun), Hidarb (Hid), Afar (Afa), Saho (Sah), Bilen (Bil), Tigre (Tgr), Tigrigna (Tig), Rashaida (Rsh), Nilotics (Nil), Beja (Bej), Ethiopians(Eth), Egyptians (Egy), Moroccans (Mor), Southern Africans (Sth), Pygmy (Pyg), Saudi Arabia (Sdi), Asia (Asi), Europe (Eur), Native Americans (NA), Australians (Ast), Nubians (Nub), Nuba (Nba) (TIF) [file pone.0097674.s004.tif]

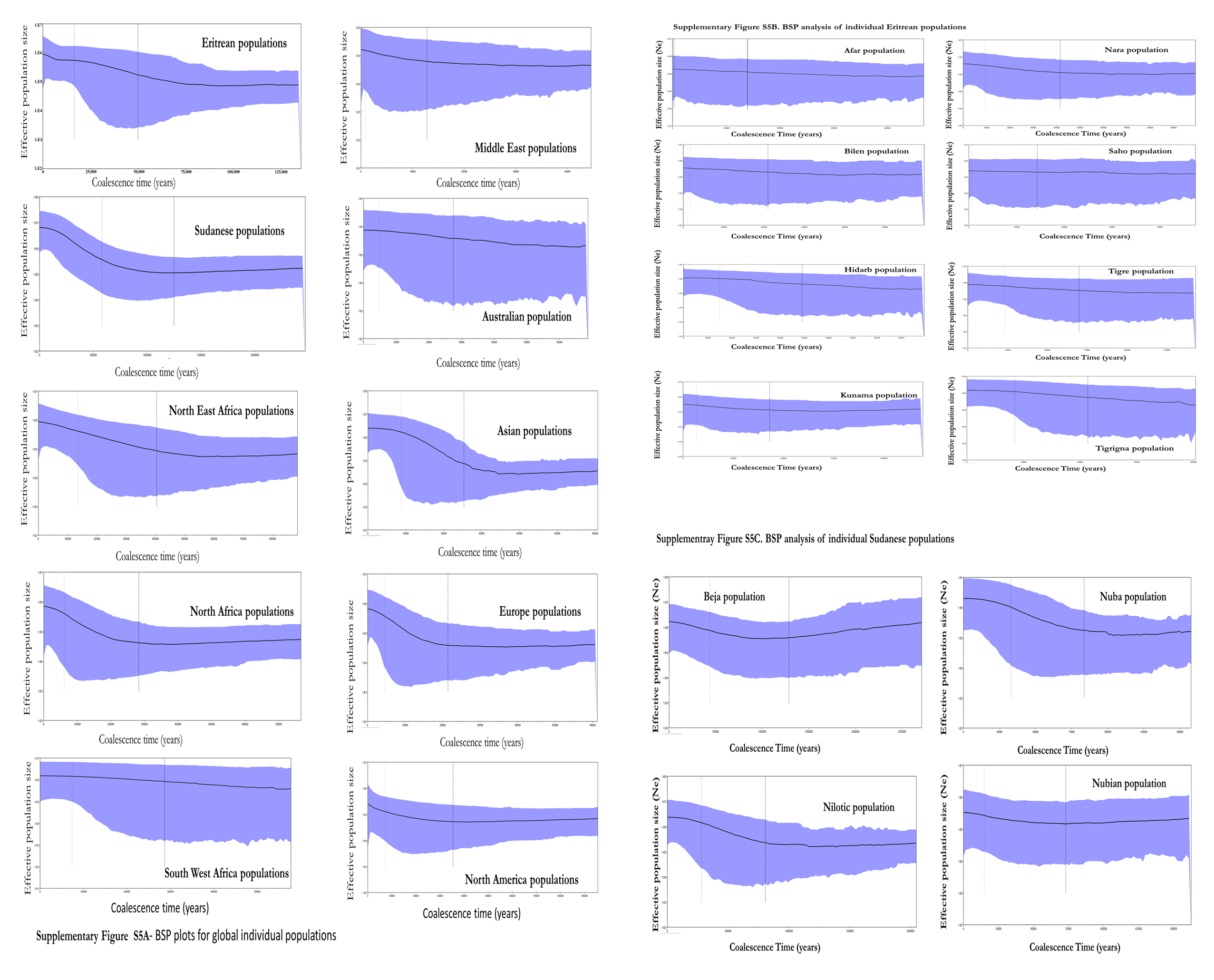

Supplement: Figure S5 — Bayesian Skyline Plots (BSP). BSP for individual population to clarify the demographic events each populations. A. Global populations, B. Eritrean populations and C. Sudanese populations. (TIF) [file pone.0097674.s005.tif]
