# Supplementary material for: The Episode of Genetic Drift Defining the Migration of Humans out of Africa Is Derived from a Large East African Population Size
Source: PLoS One. 2014 May 20;9(5):e97674. doi: 10.1371/journal.pone.0097674 (PMC4028218; doi:10.1371/journal.pone.0097674)
Supplement: Table S2 — Frequencies of 68 MT-CO2 haplotypes resolved within continental populations, the haplotype Number 1 include 164 individuals from all world populations. The rest, shared or unique haplotypes shows the name of the group possessing the haplotype. (PPTX) [file pone.0097674.s007.pptx]

## Slide 1
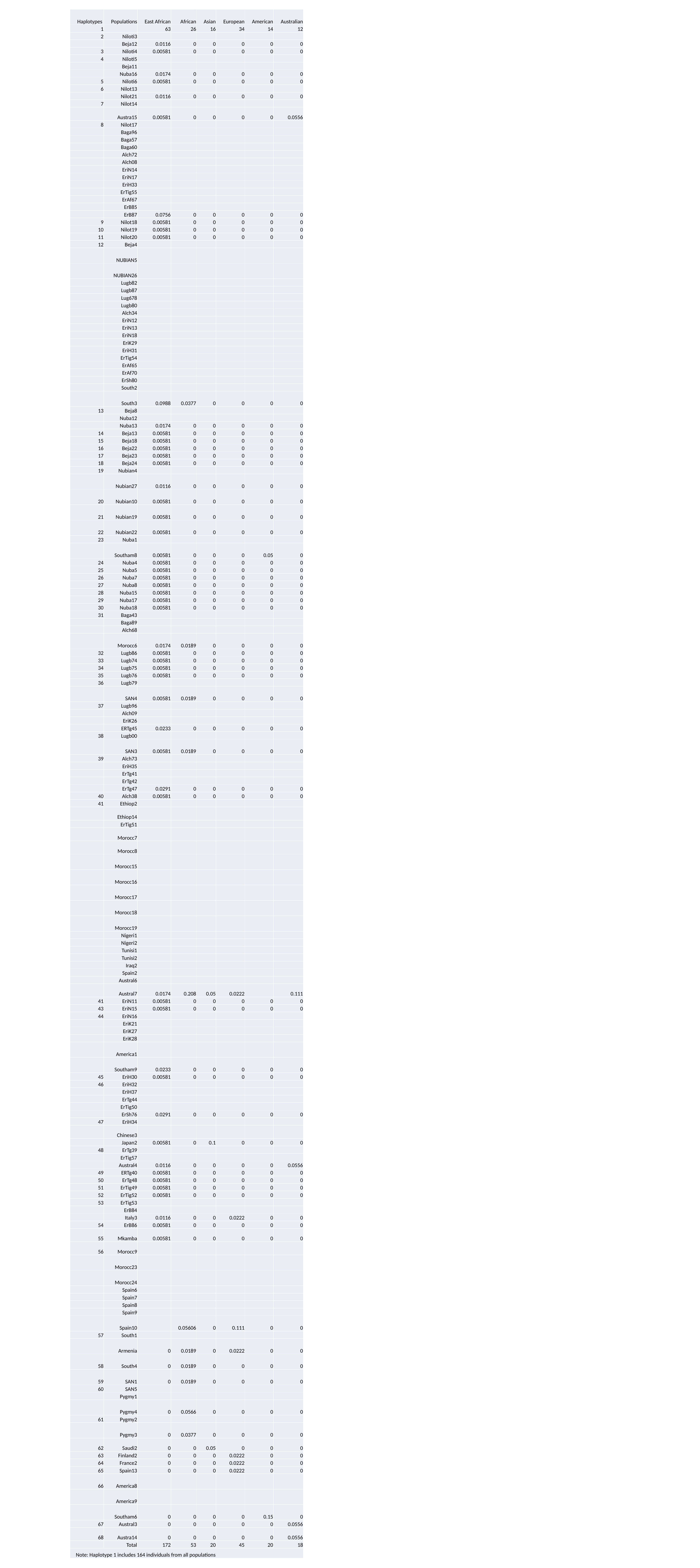

| Haplotypes | Populations | East African | African | Asian | European | American | Australian |
| --- | --- | --- | --- | --- | --- | --- | --- |
| 1 | | 63 | 26 | 16 | 34 | 14 | 12 |
| 2 | Niloti3 | | | | | | |
| | Beja12 | 0.0116 | 0 | 0 | 0 | 0 | 0 |
| 3 | Niloti4 | 0.00581 | 0 | 0 | 0 | 0 | 0 |
| 4 | Niloti5 | | | | | | |
| | Beja11 | | | | | | |
| | Nuba16 | 0.0174 | 0 | 0 | 0 | 0 | 0 |
| 5 | Niloti6 | 0.00581 | 0 | 0 | 0 | 0 | 0 |
| 6 | Nilot13 | | | | | | |
| | Nilot21 | 0.0116 | 0 | 0 | 0 | 0 | 0 |
| 7 | Nilot14 | | | | | | |
| | Austra15 | 0.00581 | 0 | 0 | 0 | 0 | 0.0556 |
| 8 | Nilot17 | | | | | | |
| | Baga96 | | | | | | |
| | Baga57 | | | | | | |
| | Baga60 | | | | | | |
| | Alch72 | | | | | | |
| | Alch08 | | | | | | |
| | EriN14 | | | | | | |
| | EriN17 | | | | | | |
| | EriH33 | | | | | | |
| | ErTig55 | | | | | | |
| | ErAf67 | | | | | | |
| | ErB85 | | | | | | |
| | ErB87 | 0.0756 | 0 | 0 | 0 | 0 | 0 |
| 9 | Nilot18 | 0.00581 | 0 | 0 | 0 | 0 | 0 |
| 10 | Nilot19 | 0.00581 | 0 | 0 | 0 | 0 | 0 |
| 11 | Nilot20 | 0.00581 | 0 | 0 | 0 | 0 | 0 |
| 12 | Beja4 | | | | | | |
| | NUBIAN5 | | | | | | |
| | NUBIAN26 | | | | | | |
| | Lugb82 | | | | | | |
| | Lugb87 | | | | | | |
| | Lug678 | | | | | | |
| | Lugb80 | | | | | | |
| | Alch34 | | | | | | |
| | EriN12 | | | | | | |
| | EriN13 | | | | | | |
| | EriN18 | | | | | | |
| | EriK29 | | | | | | |
| | EriH31 | | | | | | |
| | ErTig54 | | | | | | |
| | ErAf65 | | | | | | |
| | ErAf70 | | | | | | |
| | ErSh80 | | | | | | |
| | South2 | | | | | | |
| | South3 | 0.0988 | 0.0377 | 0 | 0 | 0 | 0 |
| 13 | Beja8 | | | | | | |
| | Nuba12 | | | | | | |
| | Nuba13 | 0.0174 | 0 | 0 | 0 | 0 | 0 |
| 14 | Beja13 | 0.00581 | 0 | 0 | 0 | 0 | 0 |
| 15 | Beja18 | 0.00581 | 0 | 0 | 0 | 0 | 0 |
| 16 | Beja22 | 0.00581 | 0 | 0 | 0 | 0 | 0 |
| 17 | Beja23 | 0.00581 | 0 | 0 | 0 | 0 | 0 |
| 18 | Beja24 | 0.00581 | 0 | 0 | 0 | 0 | 0 |
| 19 | Nubian4 | | | | | | |
| | Nubian27 | 0.0116 | 0 | 0 | 0 | 0 | 0 |
| 20 | Nubian10 | 0.00581 | 0 | 0 | 0 | 0 | 0 |
| 21 | Nubian19 | 0.00581 | 0 | 0 | 0 | 0 | 0 |
| 22 | Nubian22 | 0.00581 | 0 | 0 | 0 | 0 | 0 |
| 23 | Nuba1 | | | | | | |
| | Southam8 | 0.00581 | 0 | 0 | 0 | 0.05 | 0 |
| 24 | Nuba4 | 0.00581 | 0 | 0 | 0 | 0 | 0 |
| 25 | Nuba5 | 0.00581 | 0 | 0 | 0 | 0 | 0 |
| 26 | Nuba7 | 0.00581 | 0 | 0 | 0 | 0 | 0 |
| 27 | Nuba8 | 0.00581 | 0 | 0 | 0 | 0 | 0 |
| 28 | Nuba15 | 0.00581 | 0 | 0 | 0 | 0 | 0 |
| 29 | Nuba17 | 0.00581 | 0 | 0 | 0 | 0 | 0 |
| 30 | Nuba18 | 0.00581 | 0 | 0 | 0 | 0 | 0 |
| 31 | Baga43 | | | | | | |
| | Baga89 | | | | | | |
| | Alch68 | | | | | | |
| | Morocc6 | 0.0174 | 0.0189 | 0 | 0 | 0 | 0 |
| 32 | Lugb86 | 0.00581 | 0 | 0 | 0 | 0 | 0 |
| 33 | Lugb74 | 0.00581 | 0 | 0 | 0 | 0 | 0 |
| 34 | Lugb75 | 0.00581 | 0 | 0 | 0 | 0 | 0 |
| 35 | Lugb76 | 0.00581 | 0 | 0 | 0 | 0 | 0 |
| 36 | Lugb79 | | | | | | |
| | SAN4 | 0.00581 | 0.0189 | 0 | 0 | 0 | 0 |
| 37 | Lugb96 | | | | | | |
| | Alch09 | | | | | | |
| | EriK26 | | | | | | |
| | ERTg45 | 0.0233 | 0 | 0 | 0 | 0 | 0 |
| 38 | Lugb00 | | | | | | |
| | SAN3 | 0.00581 | 0.0189 | 0 | 0 | 0 | 0 |
| 39 | Alch73 | | | | | | |
| | EriH35 | | | | | | |
| | ErTg41 | | | | | | |
| | ErTg42 | | | | | | |
| | ErTg47 | 0.0291 | 0 | 0 | 0 | 0 | 0 |
| 40 | Alch38 | 0.00581 | 0 | 0 | 0 | 0 | 0 |
| 41 | Ethiop2 | | | | | | |
| | Ethiop14 | | | | | | |
| | ErTig51 | | | | | | |
| | Morocc7 | | | | | | |
| | Morocc8 | | | | | | |
| | Morocc15 | | | | | | |
| | Morocc16 | | | | | | |
| | Morocc17 | | | | | | |
| | Morocc18 | | | | | | |
| | Morocc19 | | | | | | |
| | Nigeri1 | | | | | | |
| | Nigeri2 | | | | | | |
| | Tunisi1 | | | | | | |
| | Tunisi2 | | | | | | |
| | Iraq2 | | | | | | |
| | Spain2 | | | | | | |
| | Austral6 | | | | | | |
| | Austral7 | 0.0174 | 0.208 | 0.05 | 0.0222 | | 0.111 |
| 41 | EriN11 | 0.00581 | 0 | 0 | 0 | 0 | 0 |
| 43 | EriN15 | 0.00581 | 0 | 0 | 0 | 0 | 0 |
| 44 | EriN16 | | | | | | |
| | EriK21 | | | | | | |
| | EriK27 | | | | | | |
| | EriK28 | | | | | | |
| | America1 | | | | | | |
| | Southam9 | 0.0233 | 0 | 0 | 0 | 0 | 0 |
| 45 | EriH30 | 0.00581 | 0 | 0 | 0 | 0 | 0 |
| 46 | EriH32 | | | | | | |
| | EriH37 | | | | | | |
| | ErTg44 | | | | | | |
| | ErTig50 | | | | | | |
| | ErSh76 | 0.0291 | 0 | 0 | 0 | 0 | 0 |
| 47 | EriH34 | | | | | | |
| | Chinese3 | | | | | | |
| | Japan2 | 0.00581 | 0 | 0.1 | 0 | 0 | 0 |
| 48 | ErTg39 | | | | | | |
| | ErTig57 | | | | | | |
| | Austral4 | 0.0116 | 0 | 0 | 0 | 0 | 0.0556 |
| 49 | ERTg40 | 0.00581 | 0 | 0 | 0 | 0 | 0 |
| 50 | ErTg48 | 0.00581 | 0 | 0 | 0 | 0 | 0 |
| 51 | ErTig49 | 0.00581 | 0 | 0 | 0 | 0 | 0 |
| 52 | ErTig52 | 0.00581 | 0 | 0 | 0 | 0 | 0 |
| 53 | ErTig53 | | | | | | |
| | ErB84 | | | | | | |
| | Italy3 | 0.0116 | 0 | 0 | 0.0222 | 0 | 0 |
| 54 | ErB86 | 0.00581 | 0 | 0 | 0 | 0 | 0 |
| 55 | Mkamba | 0.00581 | 0 | 0 | 0 | 0 | 0 |
| 56 | Morocc9 | | | | | | |
| | Morocc23 | | | | | | |
| | Morocc24 | | | | | | |
| | Spain6 | | | | | | |
| | Spain7 | | | | | | |
| | Spain8 | | | | | | |
| | Spain9 | | | | | | |
| | Spain10 | | 0.05606 | 0 | 0.111 | 0 | 0 |
| 57 | South1 | | | | | | |
| | Armenia | 0 | 0.0189 | 0 | 0.0222 | 0 | 0 |
| 58 | South4 | 0 | 0.0189 | 0 | 0 | 0 | 0 |
| 59 | SAN1 | 0 | 0.0189 | 0 | 0 | 0 | 0 |
| 60 | SAN5 | | | | | | |
| | Pygmy1 | | | | | | |
| | Pygmy4 | 0 | 0.0566 | 0 | 0 | 0 | 0 |
| 61 | Pygmy2 | | | | | | |
| | Pygmy3 | 0 | 0.0377 | 0 | 0 | 0 | 0 |
| 62 | Saudi2 | 0 | 0 | 0.05 | 0 | 0 | 0 |
| 63 | Finland2 | 0 | 0 | 0 | 0.0222 | 0 | 0 |
| 64 | France2 | 0 | 0 | 0 | 0.0222 | 0 | 0 |
| 65 | Spain13 | 0 | 0 | 0 | 0.0222 | 0 | 0 |
| 66 | America8 | | | | | | |
| | America9 | | | | | | |
| | Southam6 | 0 | 0 | 0 | 0 | 0.15 | 0 |
| 67 | Austral3 | 0 | 0 | 0 | 0 | 0 | 0.0556 |
| 68 | Austra14 | 0 | 0 | 0 | 0 | 0 | 0.0556 |
| | Total | 172 | 53 | 20 | 45 | 20 | 18 |
| Note: Haplotype 1 includes 164 individuals from all populations | | | | | | | |
